# Screening of ZIKA virus infection among dengue-like illness patients with negative RT-PCR for dengue virus in Punjab – Pakistan

**DOI:** 10.12669/pjms.37.3.3369

**Published:** 2021

**Authors:** Somia Iqtadar, Thuan Huu Vo, Mehreen Mehmood, Muhammad Salman, Mamunur Rahman Malik, Faisal Masud, Nhu Nguyen Tran Minh

**Affiliations:** 1 Somia Iqtadar, King Edward Medical University, Department of Medicine Lahore, Pakistan; 2 Thuan Huu Vo, WHO Office for Eastern Mediterranean, Department of Health Emergencies, Cairo, Egypt; 3 Mehreen Mehmood, King Edward Medical University, Department of Medicine Lahore, Pakistan; 4 Muhammad Salman, National Institute of Health, Department of Pathology, Islamabad, Pakistan; 5 Mamunur Rahman Malik, WHO Office for Eastern Mediterranean, Department of Health Emergencies, Cairo, Egypt; 6 Faisal Masud, King Edward Medical University, Department of Medicine Lahore, Pakistan; 7 Nhu Nguyen Tran Minh, WHO Office for Eastern Mediterranean, Department of Health Emergencies, Cairo, Egypt

**Keywords:** Zika virus, Dengue virus, Dengue-like Illness, Pakistan

## Abstract

**Objective:**

To detect ZIKV using reverse transcription-polymerase chain reaction (RT-PCR) among clinical samples tested negative for Dengue virus (DENV) by RT-PCR in Punjab, 2016.

**Methods:**

A descriptive cross-sectional study was carried out for duration of two months. Total of 506 samples were collected within seven days from onset of illness from all over hospitals of Punjab, Pakistan of which 350 were selected simply randomly to test for presence of ZIKV by using “Trioplex Real-Time RT-PCR Assay (Trioplex)”. Cohen’s kappa coefficient (κ) and 95% confidence interval (CI) were used to assess the degree of concordance between DENV positive results of non-structural protein 1 (NS1) and IgM solid-phase enzyme immunoassay (ELISA).

**Results:**

No samples were positive for any ZIKV, DENV or Chikungunya virus (CHIKV) by Trioplex. Among the 350 samples, 26 samples were positive concordant and the degree of concordance between NS1- and IgM-ELISA was 13% and κ coefficient was -0.71 (95% CI -0.79, -0.63).

**Conclusion:**

At study time, no samples were positive for ZIKV. Strengthening laboratory capacity to confirm arboviruses for Punjab’s laboratories is warranted. Trioplex RT-PCR has 100% sensitivity so there are nominal chances of false negative results. Establishing syndromic surveillance for Zika and conducting a sero-surveillance survey for Zika in areas with high human and Aedes mosquito density are recommended in Punjab.

**List of Abbreviations:**
ZIKV: Zika virus,
DENV: Dengue virus,
CI: Confidence interval,
NS1: Non-structural protein 1,
HRP: Horseradish peroxidase,
IQR: interquartile range,
CHIKV: Chikungunya virus,
YF: Yellow fever,
JE: Japanese encephalitis,
PoE: Points of entry.

## INTRODUCTION

Zika virus (ZIKV), transmitted predominantly by *Aedes* mosquitos, has caused major outbreaks in Micronesia, French Polynesia, Brazil and elsewhere in tropic and sub-tropic areas in recent years.^1^,^2^ The proportion of asymptomatic Zika infection is high (80%) and Zika infection is potentially associated with some severe complications including microcephaly, Guillain-Barré syndrome, and Congenital Zika syndrome.^3^,^4^

The risk of importation of ZIKV to Pakistan from viraemic travellers is tangible.^2^,^5^,^6^ The *Aedes* mosquitoes are in abundance in Pakistan although no human cases of ZIKV infection have been reported so far.^7^ Many repeated outbreaks of *Aedes*-borne diseases such as Dengue and Chikungunya, however have been reported from Pakista,^8^ especially in Punjab, where number of reported dengue cases accounted for 74% of total cases in Pakistan in the period of 2006–2011,^7^ Punjab is the most populated province with about 40% of population living in urban areas.^9^ The high urban population density and abudance of competence vector may result in the high risk of local transmission following the introduction of ZIKV through travel in Punjab, Pakistan.

Suspected dengue fever (DF) cases have been increasingly reported from Punjab in recent years; however, the proportion of confirmed DF cases has been low. In 2011 and 2012, 2470 blood samples were tested in hospitals across Punjab, of which only 1225 (50%) were positive for DENV by reverse transcription-polymerase chain reaction (RT-PCR), non-structural protein 1 (NS1) or evidence of seroconversion by solid-phase enzyme linked immunosorbent assay (ELISA). In 2016, a total of 5059 samples were collected from suspected dengue cases, of which 1286 were tested positive for DENV using both serologic tests (NS1- and/or IgM-ELISA). In clinical practice in Pakistan, occasionally positive serologic samples are sent to reference laboratory for confirmation using RT-PCR. Of these 1286 positive serologic samples, 506 were negative for DENVusing RT-PCR.

There can be a serologic cross-reactivity with Zika.^10^ Our rationale was to detect ZIKV using RT-PCR among these 506 discordant clinical samples tested negative for DENV by RT-PCR in Punjab in 2016. As ZIKV and DENV belong to same class of viruses with more or less similar presentation hence, we planned to detect zika virus in DENV negative RT-PCR samples

## METHODS

A dengue-like illness was defined as a patient with presence of fever (temperature above 98.6°F) for ≥ 2 days and ≤ 10 days and two or more of the following: headache, retro-orbital pain, myalgia, arthralgia, rash, hemorrhagic manifestations (e.g., evidenced by Hess Test, petechial, maculopapular rash, gum bleeding, bruising, hematuria, and hematemesis), abdominal pain, decreased urinary output despite adequate fluid intake with supporting laboratory evidence of leucopenia (white blood cell count < 4,000 per µL) and thrombocytopenia (platelet count <100,000 per µL. We included all samples collected within seven days from symptoms onset from patients who fulfilled the inclusion criteria of a dengue-like illness, admitted to all hospitals in Punjab, 2016 and tested positive for DENV by serologic tests but negative by RT-PCR. A total of 506 samples were collected, of which 350 were selected simply randomly to test for presence of ZIKV by using RT-PCR. The 350 samples were taken from nine hospitals in Punjab, of which 279 (79.7%) samples from Holy Family Hospital Rawalpindi, 34 (9.7%) from District Headquarter Hospital Rawalpindi, 16 (4.6%) from WAPDA Hospital, 10 (2.9%) from Services Hospital, 4 (1.1%) from District Head Quarter Hospital Lahore, 3 (0.9%) from Sir Ganga Ram Hospital, 2 (0.6%) from Nawaz Sharif Social Security Hospital, 1 (0.3%) from Allied Hospital Faisalabad, and 1 (0.3%) from District Head Quarter Hospital Sargodha. These hospitals in the four cities were the epitome for treating dengue in Punjab at that time. Small setups used to refer patient to big government set ups. We excluded samples tested negative for DENV by serologic tests and/or positive by RT-PCR.

In Punjab, all positive serum samples of suspected dengue were archived and stored at ≤ -20°C in laboratories of hospitals. All hospitals with the 350 selected samples were requested to send their selected samples to King Edward Medical University (KEMU). The samples were properly labelled and transferred using cold packs in an insulated container from Punjab central laboratory to National Institute of Health (NIH) in Islamabad to detect ZIKV using RT-PCR.

DENV IgM-ELISA Kit (Calbiotech, California, USA) was used for the detection of IgM antibody to Dengue virus in human serum or plasma. Diluted patient serum was added to wells coated with purified antigen. IgM specific antibody, if present, binds to the antigen. Then substrate was added. The intensity of the color generated was proportional to the amount IgM specific antibody in the sample.

DENV NS1-ELISA (Wantai, Beijing, China) was used for the qualitative detection of DENV NS1 antigen in human serum or plasma specimens. This kit is two-step incubation, solid-phase antibody “sandwich” ELISA in which polystyrene microwell strips were pre-coated with DENV antibodies directed against the viral NS1 antigen. Then second DENV antibody conjugated to the enzyme Horseradish Peroxidase (HRP) was added. During the second incubation step, this antibody bound to the anti-DENV – DENV NS1 Ag complexes. The amount of color intensity measured was proportional to the amount of antigen captured.

DENV RNA detection was done using *ab*TES™ DEN 4 qPCR assay kit (AITbiotech, Singapore). The Assay used was Multiplex real-time PCR technology to detect and differentiate DENV serotype-1, serotype-2, serotype-3, and serotype-4 in a single reaction tube.

Trioplex Real-Time RT-PCR Assay (Trioplex, USCDC, USA) with sensitivity of 100% and specificity of 99.1% (No cross-reactivity among arboviral diseases was observed) was used to detect ZIKV.^12^

Of the 350 patients, serological, clinical and epidemiological information, and diagnostic methods e.g., gender, age, hospital admission date, date of onset of fever, dates of samples collected for serologic testing, clinical symptoms and signs, white blood cell and platelet counts and status of discharged were extracted from hospital records. Frequencies and proportions were used to describe categorical variables and measures of central tendency were used to describe continuous variables. Data were analyzed using R software (Epi, car and psych packages). Percent agreement between NS1- and IgM-ELISA and Cohen’s kappa coefficient (κ) and 95% confidence interval (CI) were calculated to assess the degree and possibility of concordance between the two serologic testing.

All members of study team were trained on data collection and laboratory techniques for RT-PCR. The study protocol was reviewed and approved by Institutional Review Board (IRB) of KEMU no.269/RC/KEMU and WHO Regional Office for the Eastern Mediterranean (EMRO). Data was collected from dashboard of Dengue Expert Advisory Group (DEAG). It is mentioned in their terms of reference that data collected can be used for the research purpose Our objective was to detect ZIKV using reverse transcription-polymerase chain reaction (RT-PCR) among clinical samples tested negative for DENV by RT-PCR in Punjab, 2016.

## RESULTS

**Fig.1:**
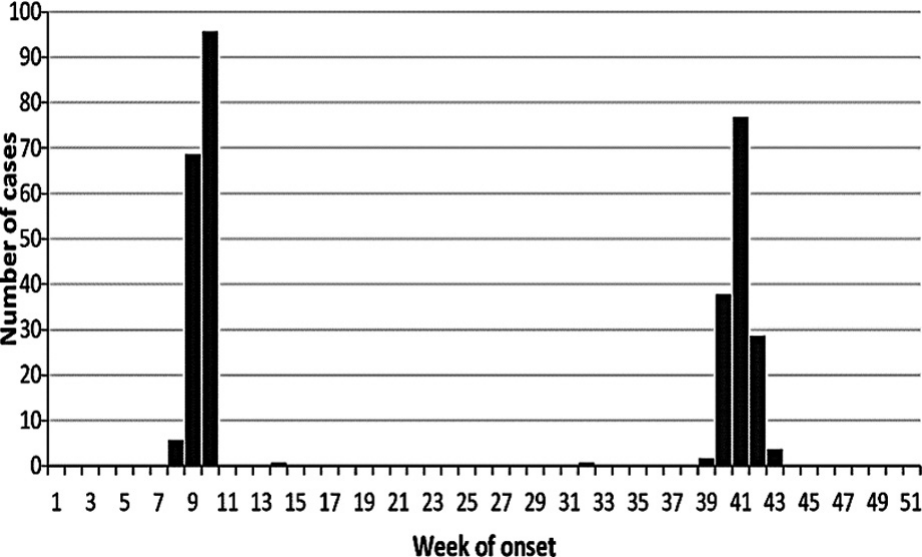
Number of cases (n=350) by week of symptom onset, Punjab, Pakistan, 2016.

**Fig.2:**
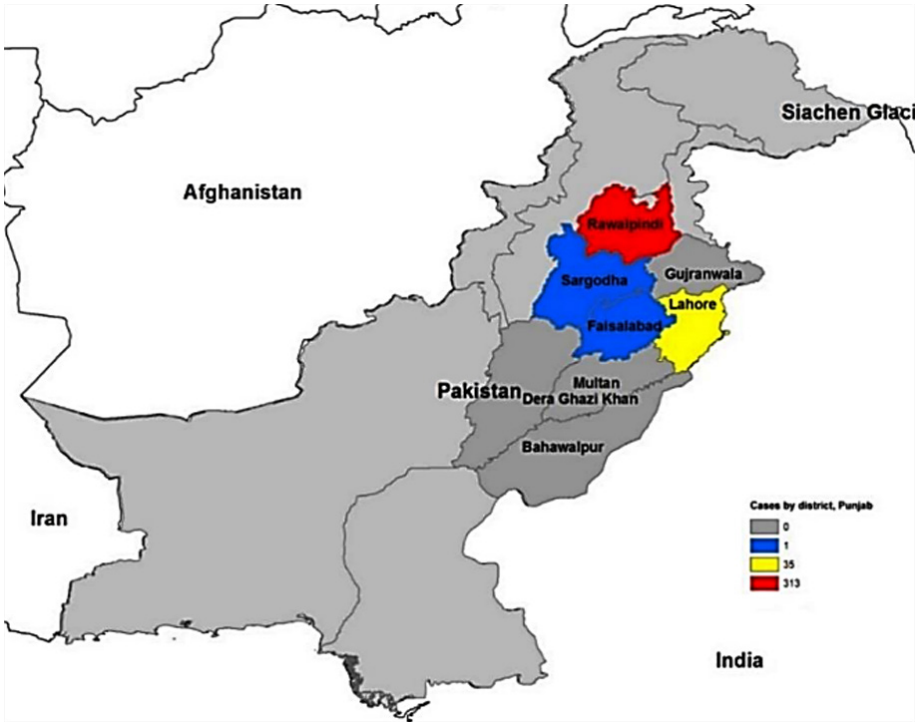
Number of cases (n=350) by district, Punjab, Pakistan, 2016.

Of 350 cases whose samples tested for ZIKV, 235 (67%) were males and the median age was 30 (interquartile range [IQR] 21–40). Most cases had onset in week 8–10 (159, 49%) and week 39–43 (150, 46%, Fig.1). Median day from date of onset to date of admission was 4 (IQR 4–5), of which 74% (258) admitted to hospital after 72 h from onset date. The cases resided in four districts of Punjab: Rawalpindi (313, 89%), Lahore (35, 10%), Faisalabad (1, 0.5%), and Sargodha (1, 0.5%, Fig.2).

The main clinical features of the 350 cases were fever (100%), headache (100%) and muscle pain (100%). Other symptoms included nausea (14%), retro-orbital pain (10%), joint pain (9%), abdominal pain (4%), vomit (3%), and hemorrhage (petechial, maculopapular rash, gum bleeding, bruising, and hematuria, 8%). Median white blood cell was 2300 per µL (IQR 1800–2800), of which 92% (322) of cases had while blood cell < 3000 per µL, and median platelet was 62,000 per µL (IQR 33,000–84,000), of which 97% (338) of cases had platelet < 100,000 per µL.

Seventy-two blood samples for NS1 and IgM (20%) were collected within 1–3 days after fever onset date while the samples were taken mainly on the fourth day (135, 39%) and the fifth day (92, 26%) from date of onset of fever with median of the fourth day (IQR 4–5). Overall, the sensitivity of NS1-ELISA decreased over time while that of IgM-ELISA increased by date of sample collected from onset. Among 154 samples positive by NS1-ELISA, 47% were on the fourth day and 89% were in days from the third to fifth from onset date while only 10% were on the first and second day from the onset of fever. Of 203 samples positive by IgM-ELISA, most positive samples were in the fourth day (36%) and the fifth day (36%), and 97% were positive in days from the third to sixth from onset date (Table-I).

**Table-I:** Positive dengue NS1- and IgM-ELISA by day of sample collected from onset of fever, Punjab, Pakistan, 2016.

Day of onset of fever	NS1-ELISA (+)		IgM-ELISA (+)	

	n (%)	Sens (%)	n (%)	Sens (%)
1st	1 (0.7)	50.0	1 (0.5)	50
2nd	9 (6.3)	100	4 (2.1)	44.4
3rd	36 (25.4)	66.7	21 (11.0)	38.9
4th	67 (47.2)	53.2	68 (35.6)	54.0
5th	23 (16.2)	24.2	69 (36.1)	72.6
6th	6 (4.2)	16.2	28 (14.7)	75.7

As some cases were positive for DENV in both tests, the sum of positive cases was higher than the total number of cases. Sens, sensitivity by sampling date

Among 154 samples positive by NS1-ELISA, 79% came from Holy Family Hospital Rawalpindi and 11% from District Headquarter Hospital Rawalpindi. Similarly to 203 samples positive by IgM-ELISA, samples from Holy Family Hospital Rawalpindi and from District Headquarter Hospital Rawalpindi were 83% and 8%, respectively (Table-II).

**Table-II:** Positive dengue NS1- and IgM-ELISA, and Cohen’s kappa co by hospitals in Punjab, Pakistan, 2016.

Hospital	NS1-ELISA (+) (n,%)	IgM-ELISA (+) (n,%)	Percent agreement	Cohen’s kappa k (95% CI)
Holy Family Hospital Rawalpindi	121 (78.6)	169 (83.3)	12.5	-0.70 (-0.79, -0.61)
District Headquarter Hospital Rawalpindi	17 (11.0)	17 (8.4)	11.8	-0.76 (-0.98, -0.55)
Wapda Hospital	8 (5.2)	7 (3.4)	6.3	-0.88 (-1, -0.64)
Services Hospital	4 (2.6)	6 (3.0)	20.0	-0.54 (-1, 0.00)
Others	4 (2.6)	4 (2.0)	27.3	-0.57 (-0.90, -0.25)
Total	154 (100)	203 (100)	12.9	-0.71 (-0.79, -063)

As some cases were positive for DENV in both tests, the sum of positive cases was higher than the total number of cases. **CI:** confidence interval; **k**, Cohen’s kappa coefficient

Of the 350 samples, the degree of concordance between NS1- and IgM-ELISA was 13%, and κ coefficient was -0.71 (95% CI -0.79, -0.63). The figures were similar in Holy Family Hospital Rawalpindi and District Headquarter Hospital Rawalpindi; the degrees of concordance between the two serologic tests were 12.5% and 11.8%, and κ coefficients were -0.70 (95% CI -0.79, -0.61) and -0.76 (95% CI -0.98, -0.55), respectively (Table-II).

Of the 350 samples tested by using Trioplex Real-Time RT-PCR Assay at NIH in Islamabad, no samples were positive for any ZIKV, DENV or CHIKV.

## DISCUSSION

The symptoms of the patients were compatible with dengue-like illness but the disease was not dengue (previously negative for DENV by RT-PCR tested in Punjab). We suspected that these patients had high probability to be infected with Zika^2^; however, we could not establish any Zika infections in this population. Although 80% samples collected within 4–6 days after illness onset, the ZIKV negative results by RT-PCR could not be explained as a decline in the level of viremia over time. It is even more difficult to explain why 20% of the remaining samples collected from 1–3 days after onset date were negative for all three viruses tested while the sensitivity of the Trioplex is 100%.^12^,^13^

After we obtained testing results by RT-PCR using Trioplex with its specificity of 99.1%, these cases were unlikely Zika, Dengue or Chikungunya.^12^ Results of previously positive DENV NS1- and IgM-ELISA are obviously false-positive which could be due to i) cross-reactivity among those who have infected with flavivirus or have vaccinated against any flavivirus diseases (e.g., yellow fever, Japanese encephalitis)^13^,^14^, ii) laboratory technical errors in Punjab, or iii) infection caused by other arboviral diseases. The patients had predominantly fever, headache and muscle pain which are less compatible with clinical pictures of yellow fever (YF) and Japanese encephalitis (JE). Furthermore, prevalence of yellow fever and Japanese encephalitis as well as the vaccination coverage of YF and JE are quite low in Punjab.^15^-^17^ Thus, the probability of false-positive because of the cross-reactivity with antibodies of YF and JE is unlikely.

The degree of concordance between the NS1- and IgM-ELISA was rather poor although the discordance of the two tests during the first week from onset is acceptable.^18^ However, the negative value of the κ coefficient may indicate that the marginal probabilities for the two serologic tests are different and probabilities to have same results between the two serologic tests in the hospitals are various (no concordance).^19^,^20^ In addition, the proportion of false-positive samples was quite high at laboratories of the hospitals. This suggests that a quality management system for the laboratories particularly in Holy Family Hospital Rawalpindi (90% false positive samples) and from Rawalpindi District Headquarter Hospital should be established or reviewed.

The clinical features of these cases were of acute viral illness. The two increases of these cases (week 8–10 and 39–43, Fig.1) occurred during the monsoon periods in Rawalpindi district (2016) are possibly due to a mosquito-borne disease. Testing results by RT-PCR (Trioplex) confirmed that these cases were not Zika, Dengue, or Chikungunya. It can be postulated that there may be another dengue-like illness whose causative agent has not yet been detected in Rawalpindi district, Punjab. A comprehensive review of these cases at hospitals in Rawalpindi district should be conducted to find out a plausible diagnosis.

Although ZIKV infection could not be established in these patients with dengue-like illness living areas with abundance of competence *Aedes*-vectors, Pakistan is at risk of introduction of Zika.^2^ In addition, a negative RT-PCR test result does not rule out infection with ZIKV.^13^ Zika, Dengue and Chikungunya have the same vectors*(Aedes aegypti* and *Aedes albopictus)* while current vector control measures (chemical/biologic use and removal of breeding sites) have failed to control virus transmission and innovative control measures are not yet established. Together with rapid urbanization in Pakistan, once viral burden in humans, Zika control poses “a particular challenge to national public health authorities because of their complex nature requiring multidisciplinary competencies and strong rapid interaction among committed sectors”.^10^ Compared with Dengue and Chikungunya, complications potential associated with Zika could result in long-term burdens on public health and economic losses. Therefore, control the introduction of ZIKV at international points of entry (PoE) and establishing syndromic surveillance for Zika are needed in Pakistan where without evidence of ZIKV cases but presence of ZIKV transmission vector.^21^,^22^

In this study, we selected a well-defined population with high probability to be infected with ZIKV and used the advanced laboratory method to detect Zika in quality samples; therefore, we consider the findings of the study are valid.

### Limitations of the study:

We used secondary data extracted from hospital records that did not provide sufficient information e.g., epidemiological and clinical history to be clearly comprehensible the laboratory testing results related to the study. Currently, medical/public health laboratories in Punjab have limited capacity to confirm the presence of any arboviruses. This could contribute to delays in identifying new public health threats.

The blood samples used to detect ZIKV in this study were of more severe cases who were admitted to hospitals while most Zika cases are mild or asymptomatic. Thus, Zika infection in community, particularly in high human and *Aedes* mosquito density areas in Punjab, is in question and the risk of local transmission of ZIKV from an infected traveller remains high in Pakistan. Raising awareness among those who travel to and from Zika endemic areas, conveyance disinfection and surveillance activities at international PoE need to be maintained.^23^

Lessons learned from spreading of the *Aedes* mosquitoes as well as of Dengue and Chikungunya within and between countries should be considered in the case of Zika in Pakistan. Increasing travel, urbanization, global trade and currently ineffective vector control measures are keys factors which should be taken into account in efforts to enhance prevention and control of Zika and arboviral diseases.^22^ Additional studies to determine the etiology of acute febrile illness are recommended once the Dengue or Chikungunya is ruled out.

## CONCLUSION

Although this study was a snapshot of screening a population with high probability to be infected with Zika, we could not establish infection in this population. Trioplex RT-PCR has 100% sensitivity so there are nominal chances of false negative results. Strengthening laboratory capacity to confirm arboviruses for Punjab’s laboratories is warranted. Establishing syndromic surveillance for Zika and conducting a sero-surveillance survey for Zika in areas with high human and *Aedes* mosquito density are recommended in Punjab.

### Authors’ Contributions:

**SI & MM** contributed to conception, design, data collection the manuscript. **THV** contributed to data management, analysis and interpretation, drafting and revising the manuscript. **MS & FM** contributed to analysis of patients’ samples. **NNTM &** **MRM** contributed to conception, design, critically revising the manuscript, and final approval of the version. **SI** accountable for the accountable of the study. All authors have read and approved the manuscript.
